# Creating Synergies between Citizen Science and Indigenous and Local Knowledge

**DOI:** 10.1093/biosci/biab023

**Published:** 2021-04-28

**Authors:** Maria Tengö, Beau J Austin, Finn Danielsen, Álvaro Fernández-Llamazares

**Affiliations:** Stockholm University and senior advisor, SwedBio, Stockholm, Sweden; Charles Darwin University, Darwin, Northern Territory, Australia; Nordic Foundation for Development and Ecology, Copenhagen, Denmark; University of Helsinki, Helsinki, Finland

**Keywords:** multiple evidence base, ecosystem management, participation, weaving knowledge systems, coproduction of knowledge

## Abstract

Citizen science (CS) is receiving increasing attention as a conduit for Indigenous and local knowledge (ILK) in ecosystem stewardship and conservation. Drawing on field experience and scientific literature, we explore the connection between CS and ILK and demonstrate approaches for how CS can generate useful knowledge while at the same time strengthening ILK systems. CS invites laypersons to contribute observations, perspectives, and interpretations feeding into scientific knowledge systems. In contrast, ILK can be understood as knowledge systems in its own right, with practices and institutions to craft legitimate and useful knowledge. Such fundamental differences in how knowledge is generated, interpreted, and applied need to be acknowledged and understood for successful outcomes. Engaging with complementary knowledge systems using a multiple evidence base approach can improve the legitimacy of CS initiatives, strengthen collaborations through ethical and reciprocal relationships with ILK holders, and contribute to better stewardship of ecosystems.

T**o widen potential sources of relevant knowledge in** use for ecosystem management, there is increasing recognition of the values and roles of knowledge-making actors beyond conventional research institutions in a range of local to global context (Bonney et al. 2014, Tengö et al. 2014, McKinley et al. 2017). In the field of ecosystem management and conservation, there has long been advocacy for engagement with local actors along the following lines: strengthening public engagement with environmental issues and building partnerships for better governance (Visseren-Hamakers 2013, Januchowski-Hartley et al. 2016), assisting efforts to document and monitor biodiversity and natural resource use and practices in areas in which scientific data is meager at best (Butchart et al. 2015, Chandler et al. 2017, Camara-Leret and Dennehy 2019), contributing local and context specific knowledge that can improve management implementation and efficiency and also increase the capacity to transform decisions into actions that are sustained over time (Danielsen et al. 2007, 2010), acknowledging the rights and stakes of people directly affected by degrading ecosystems or by conservation interventions (Farhan Ferrari et al. 2015, Brondizio and Le Tourneau 2016), and contributing complementary and unique knowledge on ecosystem dynamic and human nature interactions over time (Gadgil et al. 1993, Gavin et al. 2015).

Although science is often seen as producing the most rigorous, accurate, and useful evidence for informing decision-making, in vast areas of the world, ecosystems are governed primarily by Indigenous peoples and local communities (IPLC) whose knowledge systems and practices are as diverse as the locations and groups from which they emanate (Brondizio and Le Tourneau 2016, Garnett et al. 2018). There is a growing body of literature that calls for better recognition of Indigenous and local knowledge (ILK) as valuable knowledge in use for research, policy, and ecosystem stewardship (e.g., Mistry and Berardi 2016, Tengö et al. 2017, Sterling et al. 2017; for definition of ILK, see the glossary in Eicken et al. 2021 [this issue]). Indigenous and local peoples’ *in situ* knowledge practices have the potential to make significant contributions to meeting contemporary sustainability challenges both locally and globally (Brondizio and Le Tourneau 2016, Johnson et al. 2016, Mistry and Berardi 2016, Fernández-Llamazares et al. 2020).

Citizen science (CS) initiatives are among the approaches getting wider recognition for engaging with local actors, including holders of ILK, in science, monitoring, and rule compliance (Pocock et al. 2017, Irwin 2018). CS has contributed to increasing the participation of laypeople (often defined as people who have not been trained in science) in science policy governance processes (Leach and Fairhead 2002). In many cases, this has been aligned with work to strengthen the recognition of ILK in conservation, resources management, and planning. To achieve these interconnected targets, both CS and ILK advocates have made concerted efforts to overcome structural barriers, such as power differences, centralization and domination of decision-making by powerful actors (e.g., Hill et al. 2015), and strengthen the respect by professional scientists for laypeoples’ truth claims (Houde 2007, Roué and Nakashima 2018, Wheeler et al. 2020). Possible cognitive barriers to inclusion of nonacademic actors and their knowledge have also been identified, such as the absence of shared worldviews that are crucial to enabling collaboration and cooperation (Berkes 2015, Austin et al. 2018). On the basis of these similar aspirations of CS and ILK advocates and the solidarity they often engender, many actors have started to see CS as a method through which IPLC can mobilize their knowledge for natural resource policy, decision-making and stewardship of land and biodiversity (Bonney et al. 2014, Danielsen et al. 2018, 2021 [this issue]).

However, a central tenet of this article is that CS and ILK represent distinct types of knowledge systems—that is, the agents, practices, and institutions that organize the production, transfer, and use of knowledge (Cornell et al. 2013). We argue that keeping this in mind is critical when designing knowledge collaborations—in particular, in the context of interaction between science and ILK systems (Tengö et al. 2017). Although there may be similarities across these systems, there are also aspects that are incommensurable (Tengö et al. 2014). As has long been discussed, different knowledge systems and their experts do not carry equal weight in designing and implementing environment and conservation interventions; scientific knowledge generally has a dominant position (Agrawal 1995, Nadasdy 1999, Wheeler et al. 2020). Furthermore, although the evidence show that ILK systems and practices contribute to protect critical biological and cultural diversity; it is also clear that the same drivers leading to diversity loss have a strong negative impact on IPLC, their well-being and capacity to govern ecosystems (IPBES 2019). Therefore, both in academic and policy circles, there is increasing recognition of the need to address not only how ILK can feed into and contribute to better science and practice of ecosystem stewardship but also the reverse: How and under what conditions can science, policy, and practice support IPLC, their knowledge systems, and their governance of ecosystems?

There are a growing number of CS projects across the world that strive for close partnerships and respectful collaborations with IPLC. The main objective of this article is to compile and further address and discuss ways for CS to contribute to support and further vitalize ILK systems. To achieve this, we elaborate on CS and ILK as distinct knowledge systems and point to key aspects to take into account in building synergies while acknowledging the differences and the power asymmetries involved. We then present and discuss a number of tools and approaches that are facilitating engagement of different actors and knowledge holders in ways that nurture a multiple evidence base (MEB) approach.

## ILK systems and practices

ILK systems involve social and ecological knowledge practices and beliefs pertaining to the relationship of living beings, including people, with one another and with their environments. Such knowledge can provide information, methods, theory, and practice for stewardship of ecosystems (Gavin et al. 2015, Berkes 2018). This definition emphasizes the systems that underpin the generation and sharing of knowledge (Tengö et al. 2017). Locally developed knowledge systems are constantly changing to meet the needs of the here-and-now contexts in which they are produced, implemented, and assessed. Communities have built and relied on these knowledge systems to (among other things) support governance of complex social–ecological systems, and they are constructed, deconstructed and revised in response to interactions of local knowledge holders and their immediate social, cultural, and environmental contexts (Berkes 2018).

Knowledge systems can be characterized using several dimensions. These include notions of what constitutes valid knowledge, rules and practices for sharing and transmitting knowledge, and the attribution of ownership (Hill et al. 2016, Game et al. 2018). The debate about similarities and differences between scientific and ILK systems is long standing (Agrawal 1995, Nadasdy 1999). We will not delve into this debate in the present article but seek only to promote the notion that they represent different knowledge systems, which may have aspects of overlap as well as incommensurability, but present relevant and complementary knowledge for biodiversity and ecosystem governance. This is the starting point of the MEB approach that has been developed to guide collaborations between knowledge systems in ways that are based on equity, transparency, and usefulness for all actors involved (Tengö et al. 2014, 2017).

With respect to governance of ecosystem management and conservation, ILK is generated and developed through close interactions with the environment that are grounded in lived experience, often through stewardship practices including selection and domestication of crops and animal breeds, hunting and harvesting, habitat management and restoration, but also cultural practices, observation, and experience (Berkes 2018, IPBES 2018). Most knowledge of the environment is constructed through recurrent observations that are made sense of using the prior knowledge of the observer, including personal experience in addition to all available information that in some cases may have been handed down over generations (Berkes 2018). Combining previous experience with here-and-now observations allows the individual knowledge maker to infer something new about the world. The new knowledge that is created is then used to inform the ongoing practices of both the individual and potentially also shared with others and embedded into decision-making processes and rules in use. Hereditary governance structures and other cultural institutions are the anchor of the knowledge and customary practices (Berkes 2018).

Berkes (2015) brings attention to the cultural context of three aspects of knowledge systems, the content, processes, and values of the knowledge systems. First, the content of a knowledge system is usually empirical and for that reason is the most easily perceived as complementary across knowledge systems and cultures. As an example, this may include the knowledge of the presence or absence and perceived health of species of particular importance—for example, as a source of food or income in local environments. However, different knowledge systems may emphasize—for example, different indicators to be observed—specific attributes of the environment that may be qualitative rather than quantitative (e.g., trends in the abundance of resources rather than absolute figures) and easily observed as part of everyday practice (Tengö et al. 2014, Sterling et al. 2017, McElwee et al. 2020).

Second, there are local knowledge processes employed to construct this knowledge that are concerned with why new knowledge is required or desired, what aspects of the environment are seen to be important or significant, how observations should be undertaken, how to discuss and make sense of observations with peers, and what implications may arise from new observations. For example, which species are observed and their assigned significance, along with subjective assessments of health, are likely to vary across knowledge systems and cultures. This has subsequent implications for the actions taken by local actors to manage local social–ecological systems and the rules and institutions that are devised.

Third, all knowledge systems are underpinned by sets of values (or beliefs) concerning local relationships with the environment and how to design actions and institutions to regulate for desirable or right order of social–ecological systems. Beliefs can be very specific to cultural contexts and may not be easily shared or understood by others. For example, the abundance and health of a species could be attributed to the ongoing practice of specific resource uses or ceremonies by a local community.

**Figure 1.**
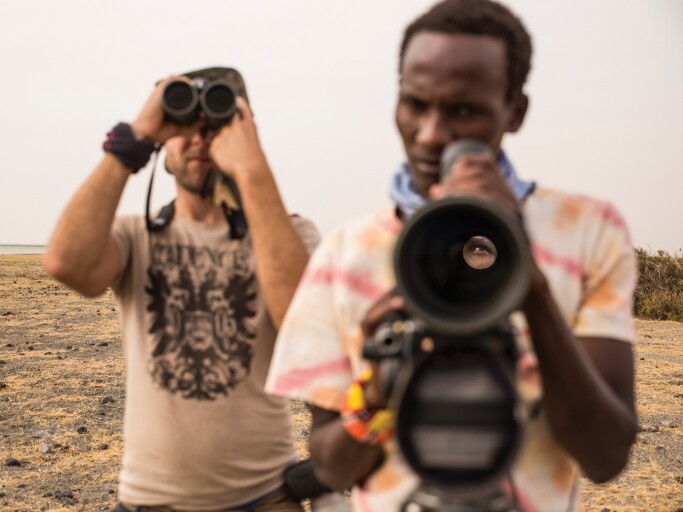
Viewpoints matter for ecosystem management and conservation. Picture from a biodiversity monitoring program among the Daasanach people of Ileret County, North Kenya. Photograph: Joan de la Malla.

Although we are elaborating in the present article on cultural aspects of ILK systems, scientific knowledge is also generated in a value context, including sources of research funding and what kind of knowledge is considered valuable in society (e.g., Levins and Lewontin 1985). However, in scientific knowledge systems there are continuous attempts to minimize the role of individuals or specific institutions or users in making observations, constructing inferences, and defining implications of the knowledge generated (Barad 2007). In contrast, ILK systems engage all aspects of human existence, including the individual knowers’ identity, culture, and beliefs. Therefore, these systems are qualitatively different from scientific knowledge systems and are fine tuned to meet diverse knowledge needs specific to cultures, worldviews, truth claim-making traditions, and institutions (figure 1).

This means that IPLC can use both scientific approaches to knowledge making, if they are perceived relevant, as well as their own local knowledge practices, and potentially weave together both approaches (Brofeldt et al. 2018, Cuyler et al. 2020). In a general sense, representatives of IPLC can be both scientists and ILK practitioners and potentially move between the two roles. However, given the intimate relationship between ILK systems and their holders and users of knowledge, it can be argued that it is more challenging for an external researcher to comprehend and relate with ILK than the other way around.

For example, Indigenous rangers in Arnhem Land, Australia are using science-based water monitoring techniques to test for salinity, toxicity, and microbiological contaminants (e.g., bacteria) in freshwater streams on their ancestral homelands. The techniques used complement local Indigenous knowledge concerning the health of waterways, such as the taste, smell, and color of water in specific places, combined with knowledge of the presence or absence of key attributes that can serve as proxies for the status and condition of freshwater ecosystems. The knowledge practices being employed to accomplish this knowledge work are diverse: local Indigenous approaches rely on holistic observations of people-places that have been known for generations, whereas the scientific techniques are much less embodied, using the objectivity of measuring instruments to create data from field sites that are turned into information through computer-based analysis. With these examples, it can be seen that to ask which information is more accurate, legitimate, or valid is to miss the point. The information produced through local Indigenous meaning-making activities is different from—although it is nonetheless complementary to—how scientific actors and activities make sense of healthy freshwater ecosystems. Both are relevant for making decisions and designing actions to adaptively manage local people-places for sustainability and conservation.

Indigenous peoples’ ways of life, including knowledge systems, ceremonies, practices, and beliefs, are protected under the United Nations Declaration on the Rights of Indigenous Peoples (UN 2008). Knowledge collaborations that helps to strengthen and enhance Indigenous peoples’ capacity to maintain their own knowledge practices makes a significant contribution to meeting obligations. Evidence of this is readily apparent in the multiple benefits currently being realized by IPLC through participation in fire management and the carbon market (Robinson et al. 2016), which has been enabled by the presence and use of ILK systems. In the following sections, we highlight how CS can support not only the maintenance and continuity of ILK systems but also the recognition of IPLC rights and customary governance.

## Citizen science: Inviting laypeople to contribute to science and enhanced environmental stewardship

On the basis of the notion that laypeople have important contributions to make to science and science-based management of the environment, CS can be described as a set of tools and approaches to invite, facilitate, and mainstream laypeople contributions into the scientific practice (Bonney et al. 2009, Miller-Rushing et al. 2012, Chandler et al. 2017). The definition used by the US Federal Crowdsourcing Act specifies that this includes enabling the formulation of research questions, creating and refining project design, conducting scientific experiments, collecting and analyzing data, interpreting the results from data, developing technologies and applications, making discoveries, and solving problems. Using a knowledge system terminology (Cornell et al. 2013), CS means engaging laypeople or volunteers as actors in a scientific (often natural science) activities, in line with the practices and rules of the academic institutions. In this setting, laypeople may represent the public—for example, any volunteer who is interested in participating­—or be engaged as experts on local conditions and trends, management practices, or particular species or resources. The latter is often the case when CS is used in the setting of IPLC (e.g., Reyes-García et al. 2020). CS is gaining momentum partly because of the possibility of gathering observation data through an interface with nonacademic actors (Dickinson et al. 2010). Moreover, CS is increasingly seen as a community engagement tool, particularly as decision-makers and land managers face increasing pressure to include citizens in conventionally top-down decision-making processes (Eitzel et al. 2017). Theobald and colleagues (2015) estimated that 1.3 million–2.3 million volunteers contribute $667 million–$2.5 billion in kind annually in biodiversity-related CS worldwide. Many CS projects include an explicit goal to contribute to enhanced environmental stewardship by supporting practices and values that are conducive to desirable biodiversity scenarios and building agency for change (e.g., Toomey and Domroese 2013, Ballard et al. 2017, McKinley et al. 2017).

Some CS projects involve citizens only in data collection—that is, contributory CS (Shirk et al. 2012). The design, analysis, and interpretation of the results are undertaken by professional scientists (e.g., Hochachka et al. 2012). Likewise, some CS projects involve volunteers in the interpretation of data only. In these projects, volunteers interpret data such as images taken by motion sensitive cameras (Curtis 2018). The volunteers visually observe photos and detect and classify specific, easily recorded features. Each classification is conducted by multiple volunteers, and the results are cross-validated. Projects such as these, in which the role of citizens is tightly limited, often involve hundreds or thousands of volunteers whose efforts are embedded within a strong organizational infrastructure that provides sophisticated professional support and feedback to the participating volunteers. The projects can use the existing engagement of citizens with the environment to collect or interpret large amounts of data that otherwise would be extremely costly for professional scientists to obtain.

Other CS projects are cocreated and involve citizens in the whole survey process—from formulation of survey questions to design, data collection, analysis, and finally use of data for natural resource management, although professional scientists may provide advice and training. Such projects are often established with the aims to contribute to fair treatment and meaningful involvement of all people regardless of origin or income with respect to the development and enforcement of laws and regulations—for instance, related to water, air, food, or human health (Rey-Mazón et al. 2018). As such, actors in the CS are often considered as data contributors and key stakeholders rather than knowledge holders *per se*. Benefits to the participants include having their voice heard, influencing how an area is managed, and learning new skills and capacities (Funder et al. 2013, McKinley et al. 2017). One example is The Extreme Citizen Science initiative (e.g., Stevens et al. 2014), that leads research practice that design and build new devices and knowledge creation processes in support of communities’ interests in different parts of the world.

In summary, CS generally represents science-based framings and practices. It includes a range of initiatives, from projects in which laypeople undertake volunteer work for science, to collaborations that provide space for meaningful engagement with IPLC concerns, perspectives, knowledge systems, and governance structures. As summarized by McKinley and colleagues (2017), care must be taken to match the needs for science and public involvement with the right type of CS project and appropriate method for involving local actors.

## Citizen science as a conduit for ILK systems

There is no doubt that CS initiatives are growing bigger, more ambitious, more diverse, and more networked all over the world (Irwin 2018). An increasing number of these initiatives are experimenting and developing new ways to involve ILK holders in CS approaches, taking steps toward deeper engagement and explicitly acknowledging the fundamental epistemic differences between science and ILK. In this section, we draw on some of this work as well as on the critique of earlier CS initiatives to elaborate on key aspirations for CS to support ILK systems and their capacity to continue to nurture biodiversity-rich ecosystems. Table 1 provides an overview, including what may be supportive practices, as well as potential risks involved when CS offers insufficient recognition of and attention to the system of actors, practices, and institutions (including values) that underpins ILK.

**Table 1.** Aspirations for supporting ILK systems and associated ecosystem stewardship, supportive actions and potential risks involved.

Aspiration	Supportive practice	Risks	Sources
ILK recognized as a valid and legitimate source of knowledge in decision-making	Recognize ILK experts and engage with and respect ILK holders as legitimate representatives of distinct epistemic traditions	Undermining legitimacy of local experts and institutions	Kimura and Kinchy 2016, Eitzel et al. 2017, Ban et al. 2018
ILK recognized as management practices, governance mechanisms, and decision support	Identify and recognize procedures and tools for generating relevant information for community decision-making	Goals, metrics and methods are externally codified and imposed on ILK holders	Pearce and Louis 2008, Housty et al. 2014, Sterling et al. 2017, Dacks et al. 2019
IPLC understanding of local social–ecological systems, including human–nature relationships, are valued and taken into account	Use participatory, collaborative and culturally appropriate methods to represent local knowledge and perspectives	Universalism (science as a superior knowledge system) hides or erases the cultural specificities of people-places relationships	Turnbull 1997, Bryan 2009, Johnson et al. 2016, Torrents-Ticó et al. 2021
Local-scale or culturally identified problems are addressed, potentially empowering local agency	Involve IPLC in identifying the topics to be addressed from the outset of the collaboration	Quality assurance and replicability are emphasized over self-determined priorities	Acharya et al. 2009, Luzar et al. 2011, Chandler et al. 2017, Wheeler et al. 2020
Knowledge governance is developed jointly and iteratively in mutual agreement	Implement free, prior, and informed consent iteratively throughout the initiative and develop joint protocols for knowledge sharing jointly. Support communities in assessing potential risks of sharing knowledge	IPLC loose access to and control of knowledge.	CBD 2004, 2011, Hill et al. 2020, Wheeler et al. 2020
IPLC are embraced as knowledge, stake-, and rightsholders	Discuss and agree with representatives of IPLC on mutually agreed terms and procedures for collaboration	IPLC are expected to participate in CS as unpaid volunteers	Johnson et al. 2016, Fernández-Llamazares and Cabeza 2018

To be a legitimate and culturally appropriate conduit for inclusion of ILK, many CS initiatives are striving to find constructive ways to embrace knowledge holders from IPLC not just as actors carrying out information tasks or data collectors or as stakeholders defining research questions but, rather, as legitimate knowledge holders, respecting that their knowledge originates from different knowledge systems (Tengö et al. 2017). Such practices can support rather than undermine local authority and disrupting knowledge in use for ecosystem stewardship (Kimura and Kinchy 2016). They can also decrease the risk of misinterpretation of knowledge and compromising the integrity and local meaning, importance and value of ILK (table 1; Nadasdy 1999, Mistry and Berardi 2016, Pyhälä et al. 2016, Reyes-Garcia and Benyei 2019).

Some positive examples can be found in Australia, New Zealand, Spain, and Greenland. The Australian Government Bureau of Meteorology built a web-based project in which ILK holders from Aboriginal communities can enter their weather calendars (www.bom.gov.au/iwk; Lefale 2010). In New Zealand, the National Institute of Water and Atmospheric Research of New Zealand aims to bring together Māori ILK and practices with standard scientific methodologies of climate observations, research, assessment, and response to human induced climate change (www.niwa.co.nz/te-kūwaha; King et al. 2008). Another example is a group of Spanish researchers that in collaboration with local community stakeholders across Spain have created CONECT-e (www.conecte.es), a Wikipedia-like CS platform aiming to gather and promote the sharing and transmission of ILK (Reyes-García et al. 2020). In these projects, community-based observing networks rely on ILK holders connected via a network to provide comprehensive information through firsthand observations of a range of environmental variables (Alessa et al. 2016). In Greenland, a pilot program for building capacity of government staff to facilitate participatory processes and knowledge collaborations has been initiated by the University of the Arctic Thematic Network on Collaborative Resource Management (UArctic 2021). Such programs could serve as models for new initiatives and training programs in other regions.

The aspirations to support ILK systems can be best served by constant efforts to recognize and value the agency of IPLC in national, as well as local or international processes. Within the Convention on Biological Diversity, several Indigenous codes of ethical conduct guide full involvement of IPLC while respecting their cultural and intellectual heritage (e.g., Akwe: Kon Guidelines and The Tkarihwaié:ri Code of Ethical Conduct; CBD 2004, 2011) that also can be useful in the context of CS initiatives. The importance of respecting the customary mechanisms of community control, ownership and transmission of ILK, and explicitly recognizing IPLC rights and institutions are also keys to the success of initiatives (Pearce and Louis 2008, Tengö et al. 2017, Fernández-Llamazares and Cabeza 2018). In particular, abiding by the principle of free, prior, and informed consent to any CS initiative in relation to ILK must be central for any ethical, equitable, and fruitful partnerships with IPLC (Ban et al. 2018). Gaining consent is not a one-off process but should be a continuous process as the work develops over time.

Data ownership and intellectual property issues need to be carefully addressed to secure adequate ownership and control of knowledge by IPLC involved (Riesch and Potter 2014). Data ownership and intellectual property issues are especially important to address when citizens are collecting or providing information concerning ILK, as knowledge may be culturally embedded, sacred, and not accessible for outsiders (Farhan Ferrari et al. 2015). Scientists who work with ILK holders should discuss and mutually agree on data ownership and other intellectual property issues at the beginning of the project (Climate and Traditional Knowledges Workgroup 2014, Austin et al. 2019, Wheeler et al. 2020). This requires transparent, equitable two-way dialogue with legitimate knowledge authorities or their representatives from the local communities. The IPBES has taken great steps forward in developing guidelines for effective dialogues and an inclusive approach for engaging with ILK in its assessments (Hill et al. 2020). The global assessment provides an interesting case for moving the practices forward through relevant standards and safeguards (McElwee et al. 2020).

A recurrent challenge of many CS initiatives is the difficulty of including, addressing, or representing those knowledge claims that might not be reducible to scientific data (Leach and Fairhead 2002, Kimura and Kinchy 2016). For example, some current geographic information systems technology applications have limited potential to represent ILK and, when used uncritically, can contribute to overlooking, or devaluing concepts that are of central importance in Indigenous cultures (Pearce and Louis 2008, Hi'iaka Working Group 2011; see also Johnson et al. 2021 [this issue]). Although participatory mapping can be useful for Indigenous peoples in their claims over territories and resources, a mapping exercise that logs mainly what is deemed important from a science-based perspective can fall short in holistic recognition of Indigenous cultural identities and histories (Bryan 2009, Kimura and Kinchy 2016). Some authors have claimed that cartographic techniques and technologies often present positivist representations of space, without expressing the spatial meanings and contexts of ILK (Kelley and Francis 2005, Pearce and Louis 2008). Several initiatives have emerged in the last years to address these caveats. For example, Indigenous geographical storytelling platforms are gaining traction in the Amazon (see PPP 2020), where place-based oral histories are documented and tied to space and territory through digital mapping technologies and open-source geographical storytelling applications (e.g., ACT 2019).

Acknowledging that volunteer actors in CS may be experts in their own right means that CS initiatives face ambiguities in the terminology that is used to refer to ILK holders. References to science in different forms may serve to raise the status of the actors involved—but also detract from the recognition of their role as holders of and experts in other epistemic traditions. Eitzel and colleagues (2017) note that ILK holders are in some cases an important group of citizen scientists, although they underline that the term *science* may or may not be appropriate or acceptable to all ILK holder groups. They even point out that, in certain cultural contexts, referring to Indigenous people as *citizen scientists*, may be inadvisable because of the historical legacies of colonialism. Interestingly, some CS projects aiming to engage ILK-holders use the term *Indigenous science* (e.g., Alessa et al. 2016).

Much of the criticism of previous attempts to incorporate ILK into CS initiatives can be traced to an insufficient recognition of the place-based ILK systems (table 1). The potential risks listed in table 1 are both procedural (i.e., concerning project delivery or participation of ILK holders) and substantive (i.e., undermining ILK systems, their relevance and capacity to guide and inform local governance of ecosystems in the longer run), and therefore require careful consideration. The consequence is an underrealized potential to mobilize and nurture legitimate knowledge for better stewardship of our planet.

## Ways forward: Ideas, tools and approaches for navigating knowledge collaborations

We have identified a set of aspirations to guide practices toward more nuanced and balanced understandings of IPLC as key knowledge holders and actors (table 1). However, IPLC knowledge must be recognized and connected with other forms of knowledge in practice and policy (Tengö et al. 2017) if they are to continue and to strengthen their critical role in ecosystem stewardship (IPBES 2019).

Below, we discuss methods and approaches that can contribute to reconciliation of the risks, needs, and opportunities. We acknowledge that no single knowledge integration process or practice for synergies between knowledge systems can be applied universally. There are subjectivities related to context and aspirations, and the answers being sought within local contexts determine the appropriateness of proposed processes (Danielsen et al. 2009, 2014, Staddon et al. 2014, Berkes 2018).

**Figure 2.**
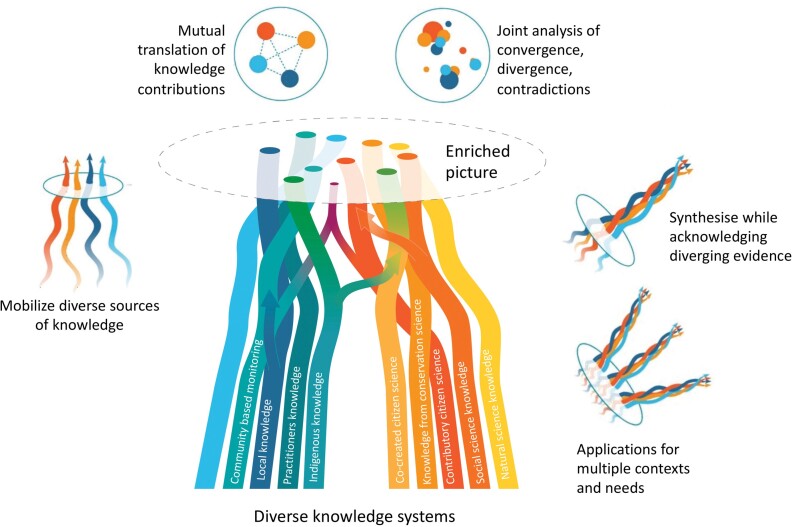
Illustration of a multiple evidence based approach to the use of knowledge for ecosystem management and conservation. Different knowledge systems are viewed as contributing complementary information and insights into a specific issue, creating an enriched picture represented by the circles in the figure. The colored strands represent contributions from different knowledge systems to the topic. Five tasks (to mobilize, translate, analyze, synthesize, and apply knowledge) provide guidance for knowledge collaborations on the basis of respect, equity among actors and knowledge systems, and usefulness for all involved. This entails engaging with actors as knowledge holders, including with the institutions and practices of generating and transmitting knowledge. They may be different than in scientific knowledge systems but nonetheless guide the generation, validity and transmission of knowledge in their respective context. Adapted from Tengö and colleagues (2014, 2017).

Tengö and colleagues (2014, 2017) present the MEB as an approach for guiding collaborations between knowledge systems. It is based on a notion of complementarity between knowledge systems and the generation of an enriched picture of a given phenomenon identified in collaboration between different stakeholders (figure 2). It depicts graphically the notion of science and other types of knowledge being weaved together to build a more comprehensive knowledge base than could be achieved by any one knowledge system alone. The MEB positions Indigenous, local, and scientific knowledge systems (among others) as different manifestations of valid and useful knowledge that generates complementary evidence for sustainable use of land areas and natural resources (see also Ban et al. 2018, Berkes 2018, Kurz and Tomaselli 2019). It has a focus of “letting each knowledge system speak for itself, within its own context, without assigning one dominant knowledge system with the role of external validator” (Tengö et al. 2014, p. 584). The MEB approach actively addresses key aspects of the knowledge systems involved: It emphasizes thoughtful engagement of actors (who is representing knowledge?), institutions (how are different ways of storing, safeguarding, and transmitting knowledge secured?), and processes (how are diverse ways for representing and engaging with knowledge employed?). The outcome can be thought of as knowledge weaving through collaborative pathways, activities, and efforts that respects the integrity of each knowledge system (Johnson et al. 2016). Malmer and colleagues (2020) reviews a number of case studies in which the MEB approach has been used in practice.

The Kimberley Indigenous Saltwater Science Project in Australia is one illustration of how a MEB can be applied in practice—and how such a process can offer an alternative pathway for understanding social–ecological systems and the often complex interaction between the landscape, ecological processes, sociocultural institutions, and economic development (box 1; Austin et al. 2019). It also illustrates how knowledge collaborations increase the legitimacy, the accuracy, and the applicability of research outcomes, potentially magnifying impacts.

**Box 1. Knowledge partnerships for saltwater country research and management.**

**Figure 3.**
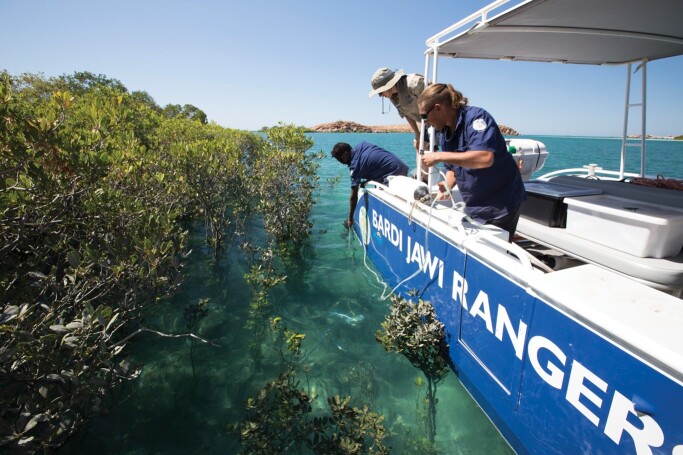
Bardi Jawi Rangers, participants in the Kimberley Indigenous Saltwater Science Project monitoring the status of the mangroves in their sea country, Australia. Photograph: Nick Thake.

The Kimberley Indigenous Saltwater Science Project (KISSP) was initiated in response to the implementation of a large, externally driven research project that sought to engage Indigenous people in producing scientific impact (www.wamsi.org.au/research-site/indigenous-knowledge). The overarching aim of KISSP was to build a regional framework for best-practice knowledge production and sharing to support state management of a network of marine protected areas across the region (figure 3).

**Figure:**
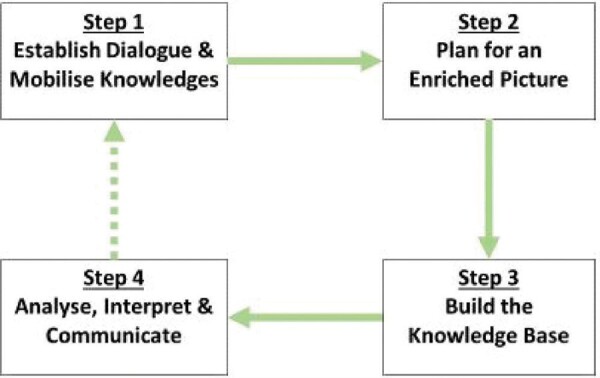

The Kimberley Science and Conservation Strategy identified that integrating Indigenous peoples’ knowledge and western science is a key element to ensuring the best outcomes for the management and conservation of the Kimberley coastal and marine environment into the future. Integration required the building of a ‘knowledge partnership’ that focused on collaboration and emphasized relationships as opposed to difference and incongruence between various kinds of knowledge. After a period of difficulties engaging the Indigenous Traditional Owners in the project (2012–2016), a forward-thinking group of local Indigenous leaders exercised their authority and brought together an Indigenous-person-led working group to govern, implement, and assess the KISSP. The group consisted of two representatives from each of the seven participating Traditional Owner groups (the Balangarra, Bardi Jawi, Dambimangari, Karajarri, Nyul Nyul, Wunambal Gaambera, and Yawuru peoples) and key staff from local Indigenous organisations.

The 14 Working Group members collectively identified research of highest priority for the collaborative management of Kimberley Saltwater Country, identified a research approach, and recruited a team of trusted researchers with whom they had experience working on numerous projects. One priority was development of a way to link local knowledge systems into a regional approach to share and weave Indigenous knowledge and western science for collaborative management of the area's natural and cultural resources. Subsequently, to facilitate the design of an Indigenous-person-led framework to guide multiple evidence based planning, the working group and research team collaboratively outlined an approach that included on-country research activities with more than 100 local Indigenous people, an online survey of scientists, and several targeted dialogue workshops. Through this collaborative process, a set of guidelines (broadly described below) has been coproduced to guide knowledge collaborations across the region.

This project has been successful in creating an ongoing, region-wide, Indigenous-person-led advisory group to provide two-way knowledge sharing, strategic advice, cooperation, and collaboration. Importantly, the advisory group does not hold any decision-making authority; this power remains solely with the individual traditional owner groups and their local governing institutions. For traditional owners and Indigenous rangers, science offers new knowledge or perspectives on changes in the country and supports enhanced decision-making for both people and country in the future. ILK–science collaborations also provide opportunities for local people to develop skills, gain employment, and then increase capabilities for influencing non-Indigenous, nonlocal organisations and institutions (e.g., governments) that can either support or hinder their aspirations. However, such benefits of knowledge integration will not flow smoothly without conscious, patient, and deliberate investment in collaborative intercultural relationships and institutions.

Step 1

- Establish and maintain meaningful dialogue.
- Assess capacities for collaboration.
- Identify goals that are mutually beneficial.
- Mobilise all knowledge systems.
- Discuss the relevance of ‘larger-than-local’ scales.

Step 2

- Collaboratively identify approach.
- Decide on a co-production or parallel integration approach
- Collaboratively identify methods.

Step 3

- Implement knowledge production in line with agreed plans.
- ‘Stick to the plan!’
- Collaboratively analyse results.

Step 4

- Collaboratively interprete results from the perspective of all stakeholders.
- Assess social, cultural, economic and environmental implications.
- Identify similarities, complementarities and/or contradictions in research outcomes.
- Collaboratively evaluate project performance.
- Jointly produce outputs and communicate results.
- Celebrate success together.

Another project, the Lion Guardians program in the Amboseli Ecosystem in Kenya provides an interesting case on how complementing conventional scientific monitoring with ILK under a contributory CS approach can help to monitor lion movements in a better informed way than scientific methods alone (Dolrenry et al. 2016). This program employs Maasai warriors as citizen scientists to collect ecological data on lion numbers and movements across community lands (Dolrenry et al. 2016, Hazzah et al. 2017). The Lion Guardians program combines science-based survey techniques (e.g., radio tracking, telemetry) with Maasai traditional tracking systems based on their ILK systems (Dolrenry et al. 2014). Each guardian patrols an area of 100 km2 that they know well, reporting lion signs, number of lions detected, age and sex of lions as interpreted from the tracks, names of the lions believed to be present, and lion predation of both wild and domestic animals (Dolrenry et al. 2016). The guardians’ knowledge and previous experience as herders and lion hunters put them in a position to follow and track lions more effectively than scientists. The engagement of Maasai knowledge holders in the research program led to an increase in the detection of lions, which resulted in an improved understanding of lions’ dispersal abilities, connectivity between populations, and the broader lion metapopulation (Dolrenry et al. 2014). In this example, the CS initiative was driven by researchers. In contrast, an example in which local citizen groups were highly involved in pushing for and designing the CS initiative is from the Achuar and Quechua peoples in the Peruvian Amazon that have led a community‐based monitoring program to map oil spills in their lands and to monitor their impacts (Cartró-Sabaté 2018; see Farhan Ferrari et al. 2015 for further examples).

The MEB approach emphasizes the role of knowledge as mobilized within knowledge systems and working directly with knowledge holders or representatives (Tengö et al. 2014, 2017). An appropriate process for doing so may differ greatly in different contexts. The literature on ILK comanagement and coproduction of knowledge includes a growing number of initiatives led by Indigenous people (Austin et al. 2019, Latulippe and Klenk 2020). Hill and colleagues (2012) present a typology of Indigenous engagement in comanagement in Australia: Indigenous-governed collaborations, Indigenous-people-driven cogovernance, agency-driven cogovernance, and agency governance. Their analysis of power sharing, participation processes, and intercultural purposes of 21 projects lead them to conclude that Indigenous governance and Indigenous-people-driven cogovernance provides better prospects for integration of ILK and scientific knowledge systems for sustainability of social–ecological systems than agency-driven cogovernance or agency governance. The Local Biodiversity Outlook reports targeting the CBD, is an interesting example of a global Indigenous-people-led initiative for monitoring biodiversity, to complement other approaches (LBO 2020). Another global example is the developing Indigenous Navigator (http://nav.indigenousnavigator.com), a framework and set of tools for and by Indigenous peoples to systematically monitor the level of recognition and implementation of their rights. Figure 1 provides an illustration of the point that is brought forward by many IPLC organizations and scholars; it matters who is looking through the binoculars.

Along the same lines is the development of Indigenous research methodologies (e.g., Denzin et al. 2008). Such initiatives are developed in response to the historical hegemony of science and the use of knowledge institutions as tools for colonization and disempowerment of IPLC. Several authors have presented this approach as a valid mechanism for recognizing and empowering ILK systems (Denzin et al. 2008, Velasquez Runk 2014, Kite and Davey 2015). This mode of academic inquiry involves holistic conceptions of social–ecological systems, including emotions and spirituality that uphold and center Indigenous practices and beliefs. Indigenous research methodologies seek to shift the focus of academic work from empirical content to an understanding and analysis of the relationships between actors, institutions, and cosmologies (Wilson 2008). These approaches highlight power differentials on the basis of factors such as race, wealth, academic status, and gender, as well as work to establish equitable ways of constructing meaning in the world through coproduction and collaboration (Tuck and Yang 2012). Box 2 illustrates how principles for an Indigenous paradigm for research programs are applied in a cocreated CS case in Greenland.

**Box 2. When is citizen science culturally appropriate? The PISUNA example.**

Useful guidance on CS and Indigenous communities might be obtained from the set of 11 principles developed for an Indigenous paradigm for research programs. In the present article, we demonstrate the use of these principles in a cocreated CS project. We describe how Greenland's Piniakkanik Sumiiffinni Nalunaarsuineq (PISUNA) locally based monitoring system (Danielsen et al. 2014, 2020) adheres to most of the principles (adapted from Pulsifer et al. 2011).

**Figure 4.**
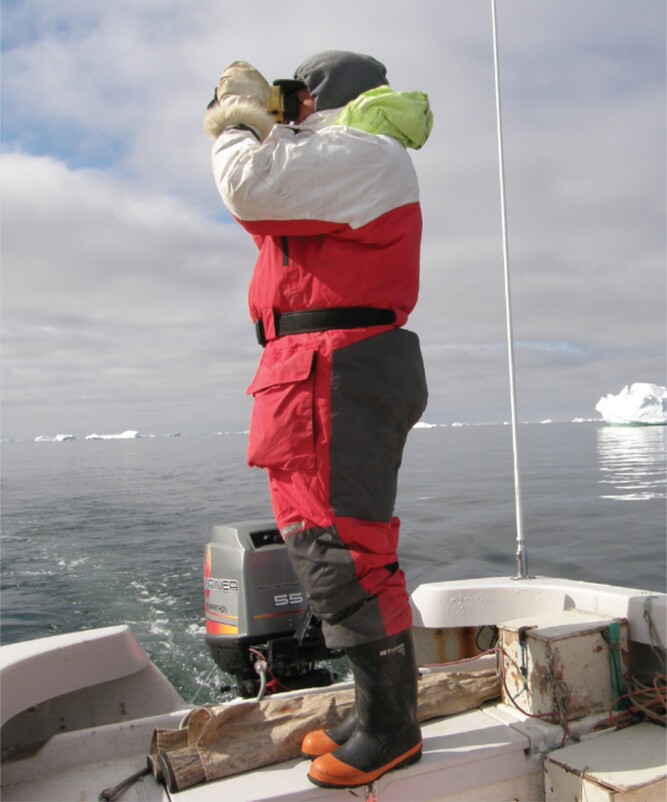
Community member scanning the sea off Disko Bay in Greenland as part of the PISUNA (Piniakkanik Sumiiffinni Nalunaarsuineq) monitoring system that builds on the local and Indigenous institutions and participants. Photograph: Martin Enghoff.

The PISUNA system was developed by Greenland's Ministry of Fisheries and Hunting with fishermen, hunters, and others to inform adaptive management of Greenland's natural resources (https://eloka-arctic.org/pisuna-net, www.pisuna.org). Natural resource committees (NRCs) were established in eight communities along Greenland's coast, including experienced fishermen and hunters and other environmentally interested people (figure 4). NRC members observe natural resources that they themselves have chosen. They meet every quarter to discuss and report their observations and proposed management decisions. Management decisions (e.g., change in quota, hunting season, gear restriction) proposed by the NRCs are presented to the Local Government Authority. The NRCs host a public meeting approximately annually. Monitoring results and decisions for the year are discussed with the entire community to validate the findings and obtain broader support for management proposals.

Is the PISUNA locally based monitoring system culturally appropriate? The local authorities’ actions, based on the NRCs’ proposals, promote respect for the observations and knowledge of the NRCs and reciprocity between different actors. Local Indigenous community members in the NRCs are taking a lead in the system. However, the democratically elected government has the option of rejecting the NRC proposals (principles 1 and 2). The system contributes to better provision for Indigenous and other local communities by encouraging a more inclusive management of natural resources (principle 3). The observations provide insights into ecological relationships (principle 4). Government staff provide feedback to the communities on their proposed management decisions, whether or not they have been acted on, and why (principle 5). The system uses Indigenous language and emphasizes oral culture (principle 6). The system attempts to make explicit who made a specific observation (principle 7). The local knowledge is given credit as a source of independent environmental information. The system thereby helps to recognize the cultural, economic, and political context of data production. The system builds on knowledge generated both by experience and direct observations but not on experimentation (principle 8). The NRC discussions and community meetings encourage open dialog and help enable the incorporation of different perspectives into the knowledge production processes (principle 9). The NRC discussions use the local context, including culturally rooted understanding of species, areas, and practices (principle 10). The interpretation of data in the system is undertaken via an inclusive and open process (principle 11). Proposals emanating from the system are, however, subject to scrutiny by the government before they can be acted on, particularly because some species are subject to international management regimes that the government has to comply with. In conclusion, the PISUNA locally based monitoring system adheres to most of the principles that make research programs culturally appropriate.

The 11 principles for an Indigenous paradigm for research programs (Pulsifer et al. 2011): 1) Respect, reciprocity and responsibility of the researchers; 2) Research designed and executed in partnership with, if not led by, Indigenous communities; 3) Research leads to a better understanding of, and provision for, Indigenous people; 4) Ontology and epistemology focus on relationships between things or ‘relationality’ (e.g. ourselves, others, environment, spirit, ideas) rather than the things themselves; 5) Researchers remain accountable for the relationships and transformations that they initiate; 6) Recognition of Indigenous languages and cultures as living processes; 7) Rejection of the notion of the objective observer – knowledge is produced in a cultural and political context; 8) The emergence of knowledge through a synthesis of experience, observation and experimentation; 9) Cooperative rather than oppositional knowledge production processes; 10) Use of metaphors and symbolism; 11) Articulating what the Indigenous research paradigm is rather than comparing with other knowledge production systems; and understanding the context of data production.

Although Indigenous-people-led approaches can maintain the integrity and value of knowledge for IPLC themselves, the challenge of building constructive interfaces with scientific knowledge systems may remain. The MEB approach provides five tasks to guide respectful collaborations between knowledge systems (figure 2; Tengö et al. 2017). The first, to mobilize, emphasizes the need to articulate local knowledge for sharing, using culturally appropriate methods. In many cases, ILK may not be visible directly as knowledge—for example, when it is embedded in various practices or aspects of everyday life, including songs and stories (see, e.g., Fernández-Llamazares and Cabeza 2018). It may also be marginalized or in decline. A process to mobilize useful knowledge and practices for environmental governance can revitalize knowledge systems, which is often a core objective in Indigenous-people-led initiatives.

Second, to translate concerns the efforts to make sure that different knowledge contributions make sense to representatives from different knowledge systems—that is, for scientific knowledge to be understandable for representatives from the local community and for local knowledge and its different dimensions to be understandable to researchers.

Thirdly, when bringing different knowledge contributions together, representatives from different knowledge systems need to be involved in analyzing and negotiating whether the contributions are overlapping, converging, or diverging. An important part is acknowledging that some aspects may be in disagreement—for example, stemming from incommensurable aspects of different knowledge systems. The last two tasks concern to synthesize and apply. To synthesize entails shaping a broadly accepted common knowledge that maintains the integrity of each knowledge system, as is illustrated in figure 2 by the braided strands, rather than integrating aspects of one knowledge system into another. When applying the knowledge, it is critical to recognize benefits and outcomes of the collaboration that can feed into different kinds of interests and needs—that is, that of local communities as well as researchers or regional decision-makers. Attention to the five tasks can guide development of CS initiatives to build on, relate with, and strengthen ILK systems.

## Conclusions

CS has contributed novel approaches for the recognition and actual incorporation of local observations, knowledge, and perspectives into decision-making processes for ecosystem stewardship. As we illustrate in this article, this has included the development of new tools and interfaces for collecting, sharing, and interpreting information across scales. The potential of further connecting CS and ILK is immense in terms of mobilizing vast knowledge in use for conservation decision-making, policy, and management; engaging more—and more diverse—individuals and communities in the science-policy process and governance more generally; implementing interventions for positive conservation outcomes more rapidly and efficiently; enhance the social legitimacy and effectiveness of CS initiatives in local settings; and realizing multiple ­benefits—environmental, as well as social, cultural, economic, and spiritual.

**Figure 5.**
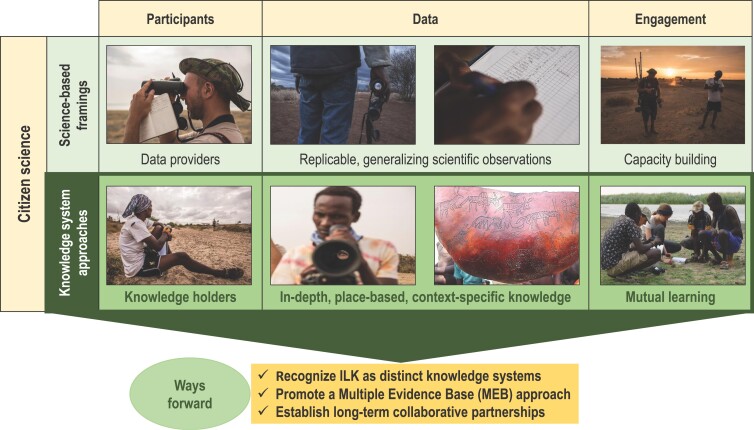
A forward looking perspective on citizen science approaches that includes both science-based and knowledge systems approaches for ecosystem management and conservation. Photographs: Biodiversity monitoring program among the Daasanach people of Ileret County, Kenya, Joan de la Malla and Álvaro Fernández-Llamazares.

ILK holders or producers offer a vast wealth of knowledge and methods that can complement scientific knowledge and further broaden and deepen the understanding of complex interactions between landscapes, ecological processes, sociocultural institutions and economic development (Ban et al. 2018, Reyes-Garcia and Benyei 2019). Collaboration with IPLC also increases the legitimacy of the generated knowledge and magnifies the potential applicability of research outcomes (Ens et al. 2016, Danielsen et al. 2020). For IPLC, science can offer new insights and perspectives and, in certain contexts, support enhanced decision-making in social–ecological systems management. ILK–science collaborations also provide opportunities for IPLC to train and develop new skills, including the ability to influence non-Indigenous or nonlocal organizations or institutions that can either support or hinder aspirations. On the basis of the examples brought forward, we posit that CS can open doors for new kinds of collaborations of comanagement and supporting diverse knowledge systems. But we also posit that attention to how, and to the processes for engagement, collaboration and partnership are of critical importance for success. In the present article, we provide some guidance and entry points for implementation, as summarized in table 1, figures 2 and 5.

A potentially critical contribution of CS for the recognition of ILK may be as a platform that contributes to making ILK more visible and relevant at a global scale. A CS approach is one way to collate multiple place-based observations and to bring ILK from local to regional or global resolutions (Eicken et al. 2021 [this issue]). Such initiatives hold promise in bringing place-based ILK into resolutions that can influence global environmental research and policy agendas (IPBES 2019, Reyes-García et al. 2019). In figure 5, we give an overview of CS as embracing science-based methods and diverse knowledge systems approaches. In this sense, the different approaches can be seen as representing different complementary streams in an MEB visualization (cf. figure 2). Recognizing these dual and potentially complementary roles of CS—as a science-based data interface that can connect local knowledge and concerns with global issues and as an emerging collaborative interface recognizing the epistemological plurality (see figure 5)—may help further development of ways forward. As we have elaborated, the view of participants, the data being generated, and the kinds of engagement (figure 5) can have strong implications for whether a CS initiative can lead to active support of ILK systems. Careful considerations should be given to which approach is appropriate in a given context—paying attention to sociopolitical factors, colonial history, and previous and current role of ILK systems in securing biodiversity, functioning ecosystems, and healthy environments.

For both the potential roles of CS proposed in figure 5—a science-based data interface and a collaborative pluralistic interface—it should be reiterated that ILK and its potential contributions to scientific or other conservation initiatives needs to be viewed in the context of human rights, claims to traditional estates and IPLC ongoing obligations or commitments to caring for the people-places they call home (Johnson et al. 2015, Kealiikanakaoleohaililani and Giardina 2016, Kutz and Tomaselli 2019). Science (including CS) is a highly relevant and useable tool for contributing to achieving these local goals, but it is only one among the many different tools being employed by IPLC to achieve locally relevant outcomes for their communities (see Fernández-Llamazares et al. 2020). Continuous dialogue with ILK holders is essential to ensure that ILK historical and contextual complexities are not overlooked in CS initiatives (Reyes-García et al. 2019, Hill et al. 2020). This includes collaboratively and iteratively designing the interfaces between knowledge systems so they can be mutually valuable and promote shared ownership of the outcomes (Tengö et al. 2017, Austin et al. 2019). Wheeler and colleagues (2020) show that projects that allow for joint, and iterative, problem definition were viewed more positively by ILK holders. The case studies from the Kimberley Indigenous Saltwater Science Project in the Kimberley, Australia (Austin et al. 2019), and the PISUNA (Piniakkanik Sumiiffinni Nalunaarsuineq) project in Greenland (boxes 1 and 2) provide examples of how such collaborations may play out in practice. There is great need for further cross-case comparison and analysis to better understand the conditions for successful implementation of MEB approaches for the benefit of all actors involved.

It is important to acknowledge that significant efforts and resources are needed to develop and maintain broad engagement with ILK holders from the outset (if not IPLC led) and collaborative partnerships over the long term. It will require adequate timeframes and attention to and tools for facilitation and mobilization of different knowledge systems, collaboration, and conflict resolution that considers rights, representation, and power dynamics (Carter 2008, Tengö et al. 2017). Although the IPBES has taken important steps forward to create space for ILK and IPLC in the processes of developing assessments (Hill et al. 2020, McElwee et al. 2020), it remains to be seen how these efforts can shift the ways that different knowledge systems are viewed and acted on in decision-making situations, including unconscious bias.

Working with multiple knowledge systems requires scientists, ILK holders, and laypeople to embrace flexible, reflexive, diverse, and at times divergent modes of making meaning and truth claims. This requires epistemological agility (Haider et al. 2018), methodological openness and, in many cases, an ability to work with dissensus so that the narratives produced can be held in tension. In doing this, space can be made for sameness and difference to be accommodated, and multiple—sometimes ­incommensurable—knowledge systems can be harnessed. Making ILK relevant beyond the local context and extending and promoting MEB approaches require that an increasing number of resource managers and scientists are able to facilitate, implement and operationalize participatory approaches and inclusive ways to generate knowledge. So far, in-service training of government staff in such techniques is very limited. Researchers wanting to engage with ILK also face hurdles in terms of lack of funding and acceptance for transdisciplinary research, including immersed and iterative methods and the demands of building long term relationships with IPLC (box 3). The time is ripe for the broad understanding of collaborative partnerships for knowledge generation in environmental stewardship to become part of the education and training of new generations of academics and practitioners—and adequately supported by funders and academic institutions.

**Box 3. The current limitations of scientific and funding institutions.**

A critical but often neglected issue is the challenges scientists face within their own science institutions and with funding agencies to engage with ILK in ecosystem management and conservation. Many scientists want to engage more fully with IPLCs, but this is precluded by the lack of support from institutions and funding agencies (Ruckelshaus et al. 2020). For example, truly honoring such work requires recognizing transdisciplinary research, immersed and iterative methods (requiring time away from institutions), long-term funding support, and accepting that results may not be quantifiable using standard publication metrics but rather by co-produced knowledge, built networks, capacity and governance development, and successful conservation actions. Scientific institutions and funding agencies need to recognize and support these pursuits. A recipe of long-term success for ILK in CS and for connecting diverse knowledge systems will not only require recognizing the needs of IPLCs and their institutions, but also raising awareness that science institutions and funding agencies have a very important part to play.

## Author Biographical

Maria Tengö (maria.tengo@su.se) is a principal researcher in Sustainability Science at the Stockholm Resilience Centre, at Stockholm University and senior advisor at SwedBio, in Stockholm, Sweden. Beau J Austin is a human geographer and is an adjunct professor at Charles Darwin University, in Darwin, Northern Territory, in Australia. Finn Danielsen is an ecologist with the Nordic Foundation for Development and Ecology, in Copenhagen, Denmark. Álvaro Fernández-Llamazares is an ethnoecologist at the Helsinki Institute of Sustainability Science, part of the University of Helsinki, in Helsinki, Finland.
